# Comment on Rose et al.: the need for responsible collection and reporting of demographic data in drug checking research

**DOI:** 10.1186/s12954-023-00913-8

**Published:** 2024-01-03

**Authors:** Lauren Airth, Trevor Goodyear

**Affiliations:** 1 British Columbia Centre On Substance Use, Vancouver, Canada; 2 Campus Wellness and Education, University of British Columbia Okanagan, Kelowna, Canada; 3 School of Nursing, University of British Columbia Okanagan, Kelowna, Canada; 4 School of Nursing, University of British Columbia, Vancouver, Canada

**Keywords:** Implementation science, Equity, Applied health research, Drug checking

We read with interest the scoping review by Rose et al. (2023), which synthesized twenty-nine articles to identify contextual factors influencing the suitability of different drug checking technologies. Several studies in this review emphasized the importance of accessibility, with service users expressing a need for services in diverse locations, including mobile options and integration into existing harm reduction services [1]. These are promising findings, especially when coupled with the authors’ determination that drug checking leaders must account for social and structural barriers to service accessibility, such as fears of criminalization, when establishing accessible drug checking services (DCS) [1]. We welcome these insights and the authors’ careful analysis of DCS implementation considerations, which are timely given the growth of DCS alongside increasingly volatile drug supplies and corresponding drug poisoning (overdose) crises in many international settings [2, 3].

We commend Rose et al. (2023) for providing a detailed account of their review strategy and approach to data extraction. Here, we were especially appreciative of the authors’ decision to extract valuable information on the drug checking technologies utilized or discussed in the included studies, as well as detail about the study designs and populations. Much of this information is presented in a Supplementary File, yet detail about the various study populations (e.g., setting, sample size, demographics) is missing from this document and is discussed in limited detail in the article itself. As researchers invested in implementation science and healthcare access equity, we see this information as essential to understanding how DCS implementation and utilization may vary across (and within) contexts and populations. Indeed, that this information is missing limits the applicability of this review's important findings on DCS, particularly since this scoping review included authors who offered meaningful data and analysis related to demographic differences [4–9]. For example, Sherman and colleagues’ survey study (2019) with N = 334 people who use drugs identified that intention to utilize DCS was associated with factors such as age, housing status, and race/ethnicity. Using a qualitative approach, another study found that some young sexual minority men were reluctant to access DCS because of concerns over safety and criminality, and felt as though such services were not “for them” (p.9) as people who used substances episodically (e.g., with sex) versus more regularly [5]. It is regrettable that considerations such as these and data about the included study populations and key demographics (e.g., age, race/ethnicity, gender) were not discussed in this scoping review.

Population considerations are critical to reflect on alongside the main themes surfaced in Rose and colleagues’ review, which underscore how other contextual factors such as venue type and legality and privacy concerns affect the implementation of DCS. These contextual issues are likely to have disproportionate impacts; it is through responsible research practices that we can identify potential differences in service utilization across populations and with respect to key demographic indices and contextual factors, such as social determinants of health and relational power dynamics. This attention toward equity is especially important in the DCS research context given the historical political, oppressive, and racist motivations of drug policies, and given that access to and experiences of healthcare—including for DCS and other harm reduction supports—continues to be shaped by social location and inequities [10–13]. Indeed, a lack of culturally informed DCS and minimal diversity among DCS staff are known barriers to service utilization [11, 14–16]. For these reasons, as suggested by Rose et al. (2023), DCS should be trauma-informed and responsive to individual and population needs.

The exclusion of demographic data in Rose and colleagues' review makes it challenging to ascertain to whom the (important) findings from the included studies may apply, and how DCS models may need to be adapted or tailored across and within populations. While Rose and colleagues conclude that the review did include studies with diverse populations, readers of the article are only prompted to delineate between two groups—'people who use drugs' and 'people who use party drugs”—throughout the authors’ analysis and the discussion of their findings. Still, Rose et al. (2023) go on to note that DCS technology is not a "one-size-fits all solution and should be considered on an individualized basis within the context of the region" (p.8). In our view, collecting and reporting information on study population and demographics is essential to understanding this context and supporting the equitable implementation of DCS.

DCS scholarship and practice are garnering more attention, and it will be prudent for all researchers to improve how demographics and potential disparities in DCS use are considered in the data we collect, interpret, and/or synthesize [10, 12, 13, 17, 18]. The need for more robust, context sensitive DCS evaluations was clearly highlighted by Rose and colleagues and this is an important contribution. Building on this call, we urge health researchers undertaking and synthesizing DCS evaluations to champion the responsible documentation of demographics and any population-specific differences that occur with respect to DCS access and other pertinent study outcomes. Likewise, when demographic data are lacking, we call on researchers to acknowledge this key limitation. In our view, the robust articulation of demographic data collected, or gaps therein, is a necessary practice to advance more equitable DCS.

## Data Availability

Not applicable.

## Abbreviation

DCS: Drug checking services
